# Genome analysis of Yucatan miniature pigs to assess their potential as biomedical model animals

**DOI:** 10.5713/ajas.18.0170

**Published:** 2018-05-31

**Authors:** Dae-Jin Kwon, Yeong-Sup Lee, Donghyun Shin, Kyeong-Hye Won, Ki-Duk Song

**Affiliations:** 1 International Agricultural Development and Cooperation Center, Chonbuk National University, Jeonju 54896, Korea; 2 Department of Animal Biotechnology, Chonbuk National University, Jeonju 54896, Korea; 3 The Animal Molecular Genetics and Breeding Center, Chonbuk National University, Jeonju 54896, Korea

**Keywords:** Yucatan Miniature Pigs, Functional Annotation, Next Generation Sequencing, Non-synonymous Single Nucleotide Polymorphisms, Genetic Association Database

## Abstract

**Objective:**

Pigs share many physiological, anatomical and genomic similarities with humans, which make them suitable models for biomedical researches. Understanding the genetic status of Yucatan miniature pigs (YMPs) and their association with human diseases will help to assess their potential as biomedical model animals. This study was performed to identify non-synonymous single nucleotide polymorphisms (nsSNPs) in selective sweep regions of the genome of YMPs and present the genetic nsSNP distributions that are potentially associated with disease occurrence in humans.

**Methods:**

nsSNPs in whole genome resequencing data from 12 YMPs were identified and annotated to predict their possible effects on protein function. Sorting intolerant from tolerant (SIFT) and polymorphism phenotyping v2 analyses were used, and gene ontology (GO) network and Kyoto encyclopedia of genes and genomes (KEGG) pathway analyses were performed.

**Results:**

The results showed that 8,462 genes, encompassing 72,067 nsSNPs were identified, and 118 nsSNPs in 46 genes were predicted as deleterious. GO network analysis classified 13 genes into 5 GO terms (p<0.05) that were associated with kidney development and metabolic processes. Seven genes encompassing nsSNPs were classified into the term associated with Alzheimer’s disease by referencing the genetic association database. The KEGG pathway analysis identified only one significantly enriched pathway (p<0.05), hsa04080: Neuroactive ligand-receptor interaction, among the transcripts.

**Conclusion:**

The number of deleterious nsSNPs in YMPs was identified and then these variants-containing genes in YMPs data were adopted as the putative human diseases-related genes. The results revealed that many genes encompassing nsSNPs in YMPs were related to the various human genes which are potentially associated with kidney development and metabolic processes as well as human disease occurrence.

## INTRODUCTION

Recently, pigs have been used in biomedical research as they share many physiological and anatomical similarities with humans. In addition, pigs are evolutionarily closer to humans than mice [1]. By generating shotgun sequence data from the pig genome, almost all ultra-conserved elements in the human genome can also be detected in pigs [1]. However, conventional pigs are much larger (>300 kg adult size) than other experimental animals (mice, rats, and dogs), which make them harder to handle. In comparison, Yucatan pigs (YMPs) are smaller than conventional pigs, with a mean body weight of 31 kg at 8 months and 83 kg at 24 months [2]. Furthermore, they have distinct traits, such as gentleness, intelligence, resistance to disease, and relative lack of odor, which make them more desirable to use for biomedical research purposes [3,4].

Genetic variations are classified by single nucleotide polymorphisms (SNPs), which occur from small insertions, deletions, conversions, and rearrangements. SNPs constitute almost 90% of the genetic variation in the human genome [5]. Most SNPs are stable and not deleterious to organisms, probably as most are located in intergenic spacers. However, non-synonymous SNPs (nsSNPs) could influence promoter activity or DNA, which is likely to affect the structure and function of proteins [6]. For instance, multiple nsSNPs in innate immunity genes can affect the susceptibility to infection, as well as the development of inflammatory disorders and autoimmune diseases [7,8]. Thus, nsSNPs are good candidates for studying disease-modifying alleles. Notably, nsSNPs that involve amino acid substitutions and cause structural and functional changes in proteins that are related to biological functions are reflective of breed characteristics [9,10].

Understanding the genetic status of YMPs and their association with human diseases will help to assess their potential as biomedical model animals. In the present study, whole genome sequencing data of YMPs was integrated and reanalyzed to identify genomic variants, and nsSNPs in selective sweep regions were identified. Using nsSNPs from YMPs, we first found deleterious nsSNPs in YMPs and then adopted these variants-containing genes in YMPs data as the putative human diseases-related genes.

## MATERIALS AND METHODS

### Sample preparation and whole genome sequencing

A whole genome sequence dataset from 12 YMPs was retrieved from the National Center for Biotechnology Information sequence read archive database (SRP047260). FastQC software (Available from: http://wwwbioinformaticsbabrahamacuk/projects/fastqc) was used to perform a quality check on raw sequence data. Trimmomatic-0.32 [11] was used to remove potential adapter sequences before sequence alignment, followed mapping of paired-end reads to the pig reference genome (*Sscrofa* 10.2.75) that was obtained from the Ensembl database using Bowtie2 [12] with default settings. Picard tools (http://broadinstitute.github.io/picard/), SAMtools [13], and Genome Analysis Toolkit (GATK) [14] were used for downstream processing and variant calling. “CreateSequenceDictionary” and “MarkDuplicates” Picard command-line tools were used to read reference FASTA sequences to write bam files with only a sequence dictionary and to filter potential PCR duplicates, respectively. Index files for the reference and bam files were created using SAMtools. Local realignment of sequence reads to correct misalignment was performed to remove small insertions and deletions using GATK “Realigner-TargetCreator” and “IndelRealigner” arguments. In addition, base quality score recalibration was performed to obtain accurate quality scores and to correct the variation in quality from machine cycle and sequence context. For variant calling, GATK “UnifiedGenotyper” and “SelectVariants” arguments were used with the following filtering criteria: all variants with i) a Phred-scaled quality score of <30; ii) read depth <5; iii) MQ0 (total count across all samples of mapping quality zero reads) >4; iv) Phred-scaled p-values of more than 200 using Fisher’s exact test were filtered out to reduce false-positive calls due to strand bias. Additionally, all filtered SNPs on autosomes (a total of 26,240,429 SNPs) were annotated using a SNP annotation tool, SnpEff version 4.1a and the Ensemble *Sus scrofa* gene set version 75 (Sscrofa10.2.75). The number of genes was 27,498.

### Identification of nsSNPs in YMP selective sweep regions

A previous study identified a total of 390 selective sweep regions and 429 associated genes in YMPs from three comparative analyses via a cross-population extended haplotype homozygosity (XP-EHH) analysis: 164 regions (184 genes), 137 regions (146 genes), and 181 regions (196 genes) in Duroc, Landrace, and Yorkshire breeds, respectively [2]. Among the 429 genes, 269 genes overlapped with nsSNP-containing genes. Thus, 269 genes containing nsSNPs were further analyzed using sorting intolerant from tolerant (SIFT) and polymorphism phenotyping V2 (Polyphen-2) to survey whether these nsSNPs were deleterious.

### Prediction of deleterious amino-acid substitutions in YMP nsSNPs

We predicted the functional effects of nsSNPs using the following *in silico* algorithms: SIFT [15] and Polyphen-2 [16]. The SIFT uses sequence homology to predict the effects of amino acid substitution on protein functions. We performed nsSNP-based sequence homology tests to identify important amino acid substitutions that might affect biological functions by structurally modifying proteins. A SIFT score of <0.05 indicated that the SNP was deleterious and could strongly affect protein function. Moreover, we performed a PolyPhen-2 (version 2.2.2) analysis using specific empirical rules to predict the effects of amino acid substitutions on the structures and functions of proteins, using amino acid sequences containing target SNPs from the Ensemble database. We then tested proteins with 1,726 nsSNPs in YMP selective sweep regions using Polyphen-2. Tested nsSNPs were classified as “probably damaging”, “possibly damaging”, or “benign” if they received Polyphen-2 scores (range: 0 to 1) of >0.95, 0.5 to 0.95, or <0.5, respectively. In this study, we predicted that “probably damaging” and “possibly damaging” SNPs would likely affect protein functions.

### Bioinformatics analysis

Genes encompassing deleterious nsSNPs in YMPs were functionally annotated and classified using the Database for Annotation, Visualization, and Integrated Discovery (DAVID) Functional Annotation Tool (http://david.abcc.ncifcrf.gov/), which provides integrated solutions for the annotation and analyses of genome-scale datasets that are derived from high-throughput technologies. The analyzed genes were considered to be able to apply to the human genes. Thus the database used in DAVID was human. We considered that gene ontology (GO) of deleterious nsSNPs and those-containing genes in YMPs might be important in human diseases, also. Additionally, pathways were elucidated according to the Kyoto encyclopedia of genes and genomes (KEGG) and the related diseases were analyzed based on the genetic association database (GAD).

## RESULTS

### DNA sequencing data preprocessing and genetic variant calling

**Figure 1:**
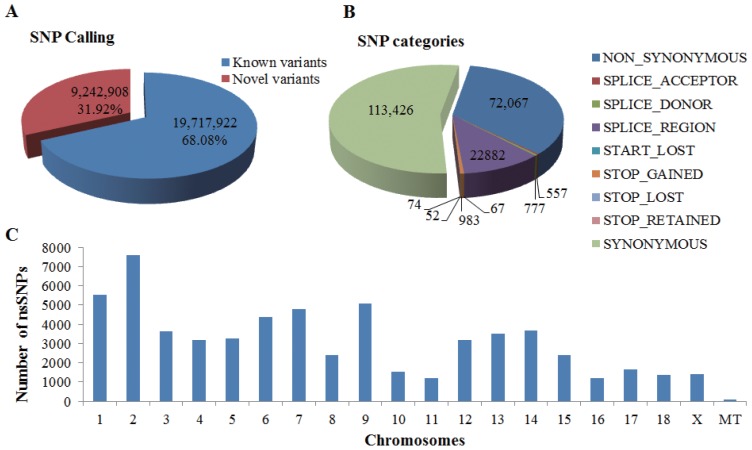
Overview of the identified single nucleotide polymorphisms (SNPs) from Yucatan miniature pigs. (A) The number of the known rs-tagged variants and novel variants. (B) The SNP categories in SnpEff analysis. (C) The number of non-synonymous SNPs across chromosomes.

We extracted a total of 28,960,830 SNPs from the whole-genome sequences of 12 YMPs and annotated all extracted SNPs using SnpEff version 4.1a [17]. The result was 8,461 genes, encompassing 72,067 nsSNPs (Figure 1). As shown in Figure 1A, the number of known variants was 19,717,922 (68.08%) and the number of novel variants was 9,242,908 (31.92%). In Figure 1B, the number of nsSNPs was 72,607 and the number of synonymous SNPs was 113,426. Thus, 72,607 nsSNPs were analyzed further to identify disease-related genes. Since YMPs are important in the investigation of human diseases, we used nsSNPs-containing genes of YMPs (8,461 genes). Figure 1C shows the number of nsSNPs per chromosome. The highest number of nsSNPs was on chromosome 2 (7,595), while the lowest number of nsSNPs was on chromosome 11 (1,202) (mitochondrial genomes were not considered).

### Selective sweep genes and genes with nsSNPs

From the 8,461 nsSNP-encompassing genes, we selected 269 genes that overlapped with the 429 selective sweep genes in the previous study [2]. Additionally, we conducted comprehensive *in silico* nsSNP analysis on YMPs and predicted the possible effects of the nsSNPs on protein structure and function using SIFT and Polyphen-2. We identified that 118 nsSNPs of 46 genes might be deleterious (Table 1). The total number of SNPs analyzed was 1,726. In the SIFT analysis, there were 224 deleterious SNPs and the number of deleterious SNPs with low confidence was 45. In the Polyphen2 analysis, there were 187 probably damaging nsSNPs, 147 possibly damaging nsSNPs, and 663 benign nsSNPs, as shown in Table 1. We selected deleterious nsSNPs in SIFT and probably damaging nsSNPs in Polyphen2 (48 in total) for GO analysis. The gene catalogue was retrieved from the ensemble website (www.ensembl.org).

### Functional classification

GO analysis was used to analyze the biological processes (BP, level 3) and KEGG pathways associated with the functioning of the genes that encompassed nsSNPs in YMPs (Table 2). GO network analysis classified 13 genes, i.e., catalytic subunit of the oligosaccharyltransferase complex, keratocan, glutamate metabotropic receptor 8 (*GRM8*), NADH: ubiquinone oxidoreductase subunit B9, dopamine receptor D5 (*DRD5*), platelet derived growth factor receptor beta (*PDGFRB*), MACRO domain containing 1, nuclear receptor corepressor 1 (*NCOR1*), follicle stimulating hormone receptor (*FSHR*), N-acylsphingosine amidohydrolase 1 (*ASAH1*), MTSS1, I-BAR domain containing (*MTSS1*), glutamyl aminopeptidase (*ENPEP*), and uroplakin 3A, encompassing 118 nsSNPs, into 5 GO terms (p<0.05) that were associated with kidney development and metabolic processes.

We identified the relationship between these genes and diseases based on the GAD and confirmed several genes with the associated disease categories. Five (ankyrin1 [*ANK1*], receptor tyrosine kinase like orphan receptor 1 [*ROR1*], early endosome antigen 1 [*EEA1*], programmed cell death 6 interacting protein [*PDCD6IP*], BMP/retinoic acid inducible neural specific 3 [*BRINP3*]), 7 (amyloid precursor protein [*APP*], *PDGFRB*, membrane palmitoylated protein 7, PHD finger protein 12, glutamate ionotropic receptor nmda type subunit 3A [*GRIN3A*], *FSHR*, arginyltransferase 1 [*ATE1*]), and 12 (*APP*, small G protein signaling modulator 1 [*SGSM1*], *ROR1*, *PDGFRB*, *GRIN3A*, seizure related 6 homolog [*SEZ6L*], *NCOR1*, *FSHR*, solute carrier family 22 member 2 [*SLC22A2*], TBC1 domain family member 32 [*TBC1D32*], OCA2 melanosomal transmembrane protein [*OCA2*], *ASAH1*) genes were associated with low-density lipoprotein (LDL) cholesterol, Alzheimer’s disease, and type 2 diabetes, respectively (Table 3).

KEGG pathway analysis identified 2 enriched pathways, hsa04080: Neuroactive ligand-receptor interaction (*GRM8*, *DRD5*, *GRIN3A*, *FSHR*) and hsa04024: cAMP signaling pathway (*DRD5*, *GRIN3A*, *FSHR*) (Table 4).

## DISCUSSION

Animal models for human disease, especially mouse models, have been used to understand the mechanisms involved in human hematopoiesis, innate and adaptive immunity, autoimmunity, infectious diseases, cancer biology, and regenerative medicine [18]. While many studies of human diseases have used mice and provided numerous insights into the biology of cancer and regenerative medicine, experiments using inbred lines of mice are limited in their translation of results to humans [19]. By comparison, pigs share many physiological, anatomical, and genomic similarities with humans. Therefore, information on the genetic status of pigs could be of greater help in the understanding of complex genetic disease variation in humans. Here, we identified nsSNPs in YMPs selective sweep regions and represented genetic nsSNP distributions that are potentially associated with disease occurrence in humans.

GO network analysis showed that 13 of 46 genes, encompassing 118 nsSNPs, were associated with kidney development and metabolic processes. Furthermore, we identified disease terms by consulting with the GAD and identified 5 genes, i.e., *ANK1*, *ROR1*, *EEA1*, *PDCD6IP*, and *BRINP3* that were associated with LDL cholesterol. LDL cholesterol is known to be a key causal factor of atherosclerotic vascular disease, especially coronary heart disease [20]. Basically, elevation of LDL cholesterol in plasma concentrations is due to single variants in the coding region of genes for the LDL receptor, apolipoprotein B, and proprotein convertase subtilisin/kexin type 9 [21]. YMPs exhibit some of the metabolic lesions observed in obese humans. Thus, insulin resistance, mild diabetes, and atherosclerotic lesions could be induced by feeding YMPs with a high-fat/high-sucrose diet [22]. In a previous study, hyperglycemia occurred in pigs that became diabetic after insulin-producing cells were destroyed, and there were no adverse effects on the kidney and liver functions [23]. Further, the lipoprotein profile in diabetic pigs was similar to that found in human diabetic patients, which suggests that pigs may be an appropriate animal model for studying metabolic disorders as they can be used to uncover the pathogenic components of the diabetic milieu or the mechanisms of atherosclerosis [24].

The genes *APP*, *SGSM1*, *ROR1*, *PDGFRB*, *GRIN3A*, *SEZ6L*, *NCOR1*, *FSHR*, *SLC22A2*, *TBC1D32*, *OCA2*, and *ASAH1*, which also encompassed nsSNPs, were classified into Alzheimer’s disease after reference to the GAD. Alzheimer’s disease is a chronic neurodegenerative disease involving loss of memory and bodily functions and ultimately causes death. Alzheimer’s disease is caused by a mutation in the *APP* gene, or in the presenilin 1 (*PSEN1*) and 2 (*PSEN2*) genes. It is now clear that mutations in all these genes alter the production of the amyloid β-peptide (Aβ) fragment of APP, and trigger the formation of amyloid plaques that eventually result in neuronal dysfunction [25]. Transgenic mice with a mutated human *APP* gene that were designed to promote Aβ accumulation and deposition formed neuronal plaques; however, neuronal loss did not occur in the hippocampus or association cortex, which is characteristic of Alzheimer’s disease [26]. As pigs are evolutionarily closer to humans, they can be used to generate transgenic pigs using a splice variant of the human APP that carries an Alzheimer’s disease-causing dominant mutation [26]. Although transgenic pigs showed high levels of transgene expression, including high levels of the corresponding protein in brain tissues, Aβ accumulation and deposition did not occur in this tissue [27]. APP processing in guinea pigs is identical to that in humans [28]. Nonetheless, there is no appropriate pig model for Alzheimer’s disease. Therefore, exploiting data for Alzheimer’s disease-related genes that encompass nsSNPs in YMPs may be useful for deciphering the mechanisms of Alzheimer’s disease and improving a therapeutic design.

## CONCLUSION

The present study surveyed the nsSNPs in YMPs and analyzed human GO using DAVID. The number of deleterious nsSNPs in YMPs was identified and then these variants-containing genes in YMPs data were adopted as the putative human diseases-related genes. The results revealed that many genes encompassing nsSNPs in YMPs were related to the various human genes which are potentially associated with kidney development and metabolic processes as well as the occurrence of human disease. Currently, the generation of transgenic pigs with single- or multiple-gene modifications, as well as chromosomal translocations, for use as biomedical models of human disease is possible using precise genetic engineering techniques [29]. With these techniques, a deeper insight into the genetic relationship between human and pig genes could help to elucidate the complex genetic disease variation-susceptibility relationship and facilitate the application of YMPs as a good candidate for a large animal model for human diseases.

## Figures and Tables

**Table 1:** Summarization of non-synonymous single amino acid variation in genes of Yucatan miniature pigs selective sweep using SIFT and Polyphen-2

Item		SIFT				

		Deleterious	Deleterious low confidence	Tolerated	Tolerated low confidence	Total
Polyphen-2	Benign	58	10	536	59	663
	Possibly damaging	47	7	81	12	147
	Probably damaging	118	7	62	0	187
	Unknown	1	21	16	21	59
	Total	224	45	695	92	1,056

SIFT, sorting intolerant from tolerant; Polyphen-2, polymorphism phenotyping v2.

**Table 2:** Gene ontology (GO) classification of genes from non-synonymous single nucleotide polymorphisms data in Yucatan miniature pigs

Term	Count	p value	Genes
GO:1901135~carbohydrate derivative metabolic process	10	0.004652	*STT3B, KERA, GRM8, NDUFB9, DRD5, PDGFRB, MACROD1, NCOR1, FSHR, ASAH1*
GO:0032835~glomerulus development	3	0.012017	*MTSS1, PDGFRB, ENPEP*
GO:0072102~glomerulus morphogenesis	2	0.020935	*MTSS1, PDGFRB*
GO:0001822~kidney development	4	0.033262	*MTSS1, PDGFRB, ENPEP, UPK3A*
GO:0055086~nucleobase-containing small molecule metabolic process	6	0.041847	*GRM8, NDUFB9, DRD5, MACROD1, NCOR1, FSHR*
GO:0072006~nephron development	3	0.051382	*MTSS1, PDGFRB, ENPEP*
GO:0048513~animal organ development	14	0.056305	*TG, MTSS1, KERA, ENPEP, UPK3A, SEZ6L, FSHR, APP, CC2D2A, ROR1, PDGFRB, TBC1D32, KAT6A, SCN10A*
GO:0007617~mating behavior	2	0.056545	*APP, DRD5*
GO:1901564~organonitrogen compound metabolic process	11	0.060428	*TG, KERA, GRM8, NDUFB9, DRD5, PDGFRB, ENPEP, MACROD1, NCOR1, FSHR, ASAH1*
GO:0021915~neural tube development	3	0.067419	*SHROOM3, CC2D2A, TBC1D32*
GO:0019098~reproductive behavior	2	0.076322	*APP, DRD5*
GO:0048731~system development	17	0.078392	*TG, MTSS1, SHROOM3, KERA, ENPEP, UPK3A, GRIN3A, SEZ6L, FSHR, APP, CC2D2A, ROR1, PDGFRB, BRINP3, TBC1D32, SCN10A, KAT6A*
GO:0007017~microtubule-based process	5	0.079313	*APP, CC2D2A, NINL, PDCD6IP, NCOR1*
GO:0034405~response to fluid shear stress	2	0.086058	*MTSS1, PDGFRB*
GO:0007618~mating	2	0.093294	*APP, DRD5*
GO:0044765~single-organism transport	14	0.09973	*TG, SERGEF, LRRC32, SLC22A23, UPK3A, GRIN3A, APP, ANK1, PDGFRB, PDCD6IP, NCOR1, SLC22A2, OCA2, SCN10A*

**Table 3:** The disease categories provided by genetic association database and disease-related genes from non-synonymous single nucleotide polymorphisms data in Yucatan miniature pigs

Term	Count	p value	Genes
Tobacco use disorder	24	1.10E-05	*TG, MTSS1, SVEP1, SHROOM3, SLC22A23, MPP7, ENPEP, SEZ6L, GRIN3A, ASAH1, ATE1, STT3B, APP, SGSM1, ANK1, GRM8, ROR1, DCLK3, PDCD6IP, BRINP3, USP43, TBC1D32, OCA2, SCN10A*
Hip	6	0.005931	*SHROOM3, ANK1, EFCAB6, PDCD6IP, BRINP3, OCA2*
Cholesterol	6	0.011794	*MTSS1, ROR1, EEA1, ENPEP, BRINP3, FSHR*
Electrocardiography	4	0.013432	*GRM8, MPP7, BRINP3, SCN10A*
Echocardiography	5	0.035536	*MTSS1, MPP7, SYNPO2, BRINP3, TBC1D32*
Body mass index	5	0.068273	*GRM8, MPP7, USP43, SLC22A2, TBC1D32*
Weight gain	3	0.068864	*GRM8, DRD5, GRIN3A*
Cholesterol, low-density lipoprotein	5	0.069947	*ANK1, ROR1, EEA1, PDCD6IP, BRINP3*
Alcoholism	5	0.081344	*MTSS1, GRM8, SYNPO2, ENPEP, SEZ6L*
Alzheimer’s disease	7	0.083627	*APP, PDGFRB, MPP7, PHF12, GRIN3A, FSHR, ATE1*
Type 2 diabetes |edema| rosiglitazone	12	0.086573	*APP, SGSM1, ROR1, PDGFRB, GRIN3A, SEZ6L, NCOR1, FSHR, SLC22A2, TBC1D32, OCA2, ASAH1*

**Table 4:** Kyoto encyclopedia of genes and genomes pathway analysis of genes from non-synonymous single nucleotide polymorphisms data in Yucatan miniature pigs

Term	Count	p value	Genes
hsa04080:Neuroactive ligand-receptor interaction	4	0.033281	*GRM8, DRD5, GRIN3A, FSHR*
hsa04024:cAMP signaling pathway	3	0.092596	*DRD5, GRIN3A, FSHR*
